# Supplementary material for: Psychometric properties of the Attitudes towards Medical Communication Scale in nursing students
Source: PeerJ. 2021 May 25;9:e11034. doi: 10.7717/peerj.11034 (PMC8162233; doi:10.7717/peerj.11034)
Supplement: Supplemental Information 2 [file peerj-09-11034-s002.docx]

**Escala Actitudes de la Comunicación Sanitaria_ Versión Española**

*Silvia Escribano, Rocío Juliá-Sanchis, Sofía García-Sanjuán, Nereida Congost-Maestre, MªJosé Cabañero-Martínez*

**Instrucciones para encuestados**:

Este cuestionario contiene 11 afirmaciones sobre las actitudes hacia a la comunicación. Marca la casilla que mejor se adapte a tu opinión, siendo:

1 = Muy en desacuerdo

2 = En desacuerdo

3 = Ni de acuerdo, ni en desacuerdo

4 = De acuerdo

5 = Muy de acuerdo

| 1. Es importante clarificar el tratamiento con los pacientes | 1 | 2 | 3 | 4 | 5 |
| --- | --- | --- | --- | --- | --- |
| 1. En general, no es necesario comprobar la comprensión de los pacientes | 1 | 2 | 3 | 4 | 5 |
| 1. La buena comunicación es una habilidad clínica básica | 1 | 2 | 3 | 4 | 5 |
| 1. Los profesionales sanitarios deben colaborar para que su trabajo sea más efectivo | 1 | 2 | 3 | 4 | 5 |
| 1. En las relaciones entre los profesionales sanitarios y los pacientes no es necesario considerar la experiencia de los pacientes sobre su enfermedad | 1 | 2 | 3 | 4 | 5 |
| 1. Tratar los problemas emocionales de los pacientes es responsabilidad de los psiquiatras, psicólogos y trabajadores sociales, no de otros profesionales sanitarios | 1 | 2 | 3 | 4 | 5 |
| 1. En la atención al paciente es importante proporcionar información sobre los hábitos de vida | 1 | 2 | 3 | 4 | 5 |
| 1. En la atención al paciente es esencial abordar sus emociones y problemas psicosociales (por ejemplo, problemas familiares, económicos, etc.) | 1 | 2 | 3 | 4 | 5 |
| 1. La buena comunicación entre los profesionales sanitarios y los pacientes mejora los resultados de salud (por ejemplo, reingresos, calidad de vida, etc.) | 1 | 2 | 3 | 4 | 5 |
| 1. La comunicación no verbal de los profesionales sanitarios no afecta, en general, a los pacientes | 1 | 2 | 3 | 4 | 5 |
| 1. En la consulta con el paciente, los profesionales sanitarios deben ser conscientes de su lenguaje corporal y de la distancia interpersonal | 1 | 2 | 3 | 4 | 5 |

**Instrucciones para investigadores:**

Los ítems 2, 5, 6 y 10 se puntúan de manera inversa (totalmente en desacuerdo = 5 y totalmente de acuerdo = 1).

La puntuación total (de 11 a 55 puntos) es la suma de los 11 ítems, donde cuanto más alta es la puntuación, más positiva es la actitud de los participantes hacia la comunicación.
